# Classic and new candidate markers for drug resistance in a large cohort of leprosy patients from the Amazon state, Brazil

**DOI:** 10.1128/aac.01550-24

**Published:** 2025-05-20

**Authors:** Cynthia de Oliveira Ferrreira, André Luiz Leturiondo, Camila Gurgel dos Santos, Jaqueline Bentes da Silva, Michelle Fernanda de Andrade Souza, Catherine Bianca Oliveira Rego, Guilherme Caldas de Souza, Thamires Bastos Pinheiro, Gisely Cardoso Melo, Patricia Sammarco Rosa, Marcelo Távora Mira, Charlotte Avanzi, Carolina Talhari

**Affiliations:** 1 Laboratório de Biologia Molecular, Fundação Hospitalar Alfredo da Matta-FUHAM, Manaus, Amazonas, Brazil; 2 Genomic Health Surveillance Network: Optimization of Assistance and Research in the State of Amazon (REGESAM), Manaus, Amazonas, Brazil; 3 Programa de Pós-Graduação em Medicina Tropical, Universidade do Estado do Amazonas–UEA 115526, Manaus, Amazonas, Brazil; 4 Instituto de Pesquisa Clínica Carlos Borborema, Fundação de Medicina Tropical Dr Heitor Vieira, Manaus, Amazonas, Brazil; 5 Laboratório de Biologia Molecular, Instituto Lauro de Souza Lima 368269, Bauru, Brazil; 6 Graduate Program in Health Sciences, School of Medicine and Life Sciences, Pontifícia Universidade Católica do Paraná 196608, Curitiba, Brazil; 7 Mycobacteria Research Laboratory, Department of Microbiology, Immunology and Pathology, Colorado State University 164597 , Fort Collins, Colorado, USA; University of California San Francisco, San Francisco, California, USA

**Keywords:** leprosy, antimicrobial resistance, DNA sequence analysis, Brazilian Amazon region

## Abstract

Multidrug therapy for leprosy is highly effective and the recommended standard of care for leprosy worldwide. However, reports of antimicrobial resistance (AMR) have emerged globally. This study aimed to estimate the frequency of primary and secondary AMR associated with leprosy in patients treated at the Alfredo da Matta Foundation, Manaus, Amazonas, Brazil, as well as to determine the circulating subtypes of *Mycobacterium leprae* in this population. A total of 315 biopsy samples were investigated for variants in leprosy AMR-associated genes (*rpoB, folP1, gyrA*); a subset of 163 samples was also investigated for 5 additional candidate genes: *gyrB, ctpC, ctpI, ribD*, and *fadD9*. Patients were categorized into new cases, relapses, and suspected treatment failures. For statistical analysis, Pearson’s chi-square or Fisher’s exact test was employed for categorical variables, while mean and SD were calculated for continuous variables, with a significance level of 5%. Variant analysis detected 10 resistant *M. leprae* isolates displaying mutations in the *rpoB* (2, 0.6%) and *folP1* (8, 2.5%) genes. In addition, variants in *gyrB* (1, 0.6%), *ctpC* (6, 3.7%), *ribD* (4, 2.4%), and *fadD9* (15, 9.2%) were detected. Nine out of 10 resistant isolates were observed in the relapse group (*P* = 0,0014). Despite the low variant frequencies observed, variant detection highlights the need for expanded antimicrobial monitoring and surveillance. The impact of mutations in *ribD* and *fadD9* on therapeutic response remains unclear, underscoring the need for further research. Genotyping revealed subtype-4 predominance (79.6%). Our findings highlight the importance of comprehensive AMR monitoring, particularly in relapse cases.

## INTRODUCTION

Leprosy is a chronic infectious disease caused by *Mycobacterium leprae* and *Mycobacterium lepromatosis,* primarily affecting the skin and peripheral nerves (1). In 2022, the World Health Organization (WHO) reported 184,087 new cases of leprosy worldwide, with India, Brazil, and Indonesia accounting for 78.1% of these cases (2). In Brazil, new cases decreased by 28.4% from 2019 to 2022, likely due to the COVID-19 pandemic (3). In Amazonas, the detection rate in 2022 was 8.8 per 100,000 inhabitants, near the national average (3).

Multidrug therapy (MDT), introduced in Brazil in the 1980s, is the standard treatment for leprosy and includes rifampicin (the only bactericidal drug) along with the bacteriostatic dapsone and clofazimine (4). Since 2021, a uniform MDT has been used, with a 6-month regimen for paucibacillary (PB) and 12-month regimen for multibacillary (MB) cases (4).

Early diagnosis and effective treatment are key to leprosy control, highlighting the importance of antimicrobial resistance (AMR) monitoring, which has been recommended by the WHO since 2009 (5). Reports of AMR in Brazil, including in the Amazon region, have prompted the establishment of the National AMR Surveillance Network in 2018, focusing on new MB cases, relapses, and suspected treatment failures (4, 6, 7). Since *M. leprae* cannot be cultured in axenic media, molecular surveillance of drug resistance-associated regions in *rpoB* (rifampicin), *folP1* (dapsone), and *gyrA* (ofloxacin) genes is the most efficient method for detecting resistance (8). Surveillance data from 2018 to 2022 revealed low AMR prevalence in Brazil (2.48%; 61/2,463) but highlighted significant multidrug-resistant strains (6.5%) and increased ofloxacin resistance (50.8%) among relapse cases, which had the highest AMR incidence (3.6%) (9). However, genes associated with clofazimine resistance remain undetermined.

These results likely underestimate the true extent of *M. leprae* resistance, as only 2,463 biopsies were analyzed compared to approximately 83,500 new cases during the same period (3). Moreover, recent studies have shown resistance in *M. leprae* strains without mutations in standard resistance genes. For example, the phenotypically multiresistant *M. leprae* Airaku strain has a wild-type *rpoB* gene, suggesting alternative resistance mechanisms (10). Whole-genome analyses of the Airaku strain revealed mutations in *ctpC* and *ctpI,* genes in the ATPase transporter gene family associated with resistance (10). Additionally, while *gyrA* gene variants are linked to quinolone resistance (11–13), mutations in the *gyrB* gene, including Asp464Asn, Asn502Asp, and Glu504Val, have been associated with ofloxacin resistance (14). A global phylogenetic study of 154 *M*. *leprae* genomes identified novel mutations in the *fadD9, ribD*, *ethA, pks4,* and *n*th genes, which are frequently hypermutated in resistant strains (15). In *M. tuberculosis*, mutations in the *rpoA* and *rpoC* genes, which encode RNA polymerase subunits, are known to compensate for *rpoB* mutations in rifampicin-resistant strains (16), but such correlations with resistant *M. leprae* resistance remain unclear (17).

A comprehensive understanding of AMR-associated gene variants is necessary to scale up resistance detection in *M. leprae* strains. This study aims to assess the prevalence of AMR in leprosy patients treated at a reference center in the Brazilian Amazon region by analyzing both classic and novel genes linked to drug resistance and identifying the circulating *M. leprae* subtypes in this population.

## MATERIALS AND METHODS

### Study design and data source

This descriptive study analyzes AMR surveillance in leprosy patients treated at the Alfredo da Matta Foundation (AMF) in Manaus, Amazonas, Brazil. Classic AMR screening of the *rpoB, folP1,* and *gyrA* genes was conducted on skin biopsy samples collected from 2012 to 2022. Screening of novel AMR candidate genes and *M. leprae* genotyping was performed on a subset of samples collected between 2012 and 2018, stored in 70% alcohol at −20°C. Clinical and laboratory data were obtained from AMF medical records and the Brazilian Notifiable Diseases Information System.

### Study population and case definition

Skin biopsies were collected from three groups: (i) new MB leprosy cases with a bacilloscopic index (BI) >2+ , (ii) relapse cases, and (iii) cases of suspected treatment failure. Leprosy diagnosis followed Brazilian Ministry of Health criteria: skin lesion(s) or sensory nerve changes, thickened peripheral nerves, and confirmation of *M. leprae* presence by skin smear or biopsy. New cases were diagnosed clinically, and *M. leprae* identification was confirmed by histopathology and skin smear. Only MB cases (BI >2+ ) were included. Relapse was defined as the reappearance of leprosy symptoms after at least 5 years of apparent cure following MDT (4). Suspected treatment failure was diagnosed in patients showing continued disease activity after completing adequate leprosy treatment. Primary resistance refers to resistant isolates of newly diagnosed patients, while secondary resistance includes cases of relapse or suspected treatment failure.

### Methods of AMR detection

Skin biopsy specimens (4 or 6 mm punch) were preserved in 70% ethanol and stored at −20°C. DNA was extracted using the DNeasy Blood and Tissue Kit (Qiagen). *M. leprae* DNA was detected via real-time PCR (qPCR) targeting the 16S rRNA gene, using human β-actin as an internal control (18).

AMR detection followed WHO guidelines, using DNA sequencing to identify single-nucleotide polymorphisms (SNP) in the *folP1* (dapsone), *rpoB* (rifampicin), and *gyrA* (ofloxacin) genes (8). PCR conditions and primers are listed in Tables S1 and S2. Amplification products were analyzed using 1.5% agarose gel electrophoresis. Sequencing reactions were performed using the BigDye Terminator v3.1 Kit (Applied Biosystems), followed by capillary electrophoresis on an ABI 3130 or SeqStudio genetic analyzer (Applied Biosystems). Mutations were identified by comparing the sequences with reference data from NCBI.

### Novel candidate genes for AMR screening

A subset of 163 samples was screened for target region mutations in the *gyrB, ctpC, ctpI, ribD,* and *fadD9* genes (10, 14, 15). Primers were designed specifically for this study (Table S1). For the *fadD9* gene, three primer sets were used to cover the 1.75 kp region containing previously reported variant sites (15).

### *M. leprae* genotyping

*M. leprae* genetic diversity was assessed using three SNP markers (SNP7614, SNP14676, and SNP2935685), as described in Table S2. These SNPs were used to assign genotypes (1–4) (19).

### Data analysis

Descriptive statistics analyzed demographic variables (sex and age) and clinical variables (case type and gene mutations). The proportion method recommended by the WHO (2) was applied. Categorical variables were compared using Pearson’s chi-square or Fisher’s exact test, while continuous variables were expressed as mean ± SD. A significance level of 5% was used for all analyses. Statistical analysis was performed using Epi Info version 7.2.5.0 (https://www.cdc.gov/epiinfo/por/pt_index.html).

## RESULTS

**Fig 1:**
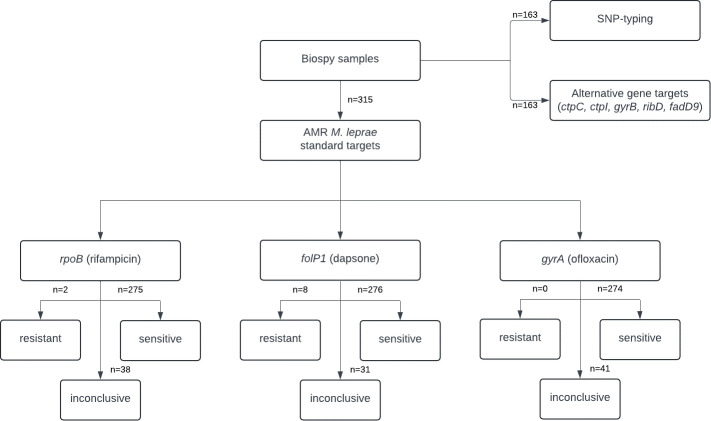
Flowchart of biopsy samples investigated for AMR and novel genes in the state of Amazonas between 2012 and 2022. The numbers outside the boxes represent the quantity of samples for each analysis.

A total of 315 biopsy samples collected between 2012 and 2022 were analyzed (Fig. 1). Of these, 103 (32.7%) were new cases, 114 (36.2%) were relapses, and 98 (31.1%) were suspected treatment failures. The average age of the patients was 47.3 years (SD: 14.9), with a predominance of males (82.5%, 260) (Table 1). Geographically, 77.1% (*n* = 243) of cases were from Manaus, while 22.9% (*n* = 72) were from neighboring states of Roraima and Pará (Table S3).

**TABLE 1:** Clinical and laboratory data of the 315 patients whose clinical samples underwent AMR investigation for leprosy at AMF^*a*^

Variable	Total (*N* = 315)	New cases (*N* = 103)	Relapse (*N* = 114)	Suspect of failure (*N* = 98)
Sex male, *n* (%)	260 (82.5%)	81 (78.6%)	93 (81.6%)	86 (87.7%)
Age, years, median (IQR)	47.3 (36–58)	45.1 (35–57)	50.4 (40–60)	46.1 (35–55)
Resistant cases	10 (3.2%)	0	9 (7.9%)	1 (1%)
*rpoB* (rifampicin)				
Resistant, *n* (%)	2 (0.6%)	0	2 (1.7%)	0
Nonresistant, *n* (%)	275 (87.3%)	86 (83.5%)	105 (92.1%)	84 (85.7%)
Inconclusive, *n* (%)	38 (12.1%)	16 (15.5%)	7 (6.1%)	14 (14.3%)
*folP1* (dapsone)				
Resistant, *n* (%)	8 (2.5%)	0	7 (6.1%)	1 (1%)
Nonresistant, *n* (%)	276 (87.6%)	90 (87.4%)	101 (88.6%)	85 (86.7%)
Inconclusive, *n* (%)	31 (9.8%)	13 (12.6%)	6 (5.3%)	14 (12.2%)
*gyrA* (ofloxacin)				
Resistant, *n* (%)	0	0	0	0
Nonresistant, *n* (%)	274 (86%)	78 (75.7%)	106 (93%)	90 (91.8%)
Inconclusive, *n* (%)	41 (13.0%)	25 (24.3%)	8 (7.0%)	8 (8.2%)

^*a*^ Data from the Molecular Biology Laboratory/AMF and the Resistance Investigation System (sirH)—MS/SVSA/DEDT.

Mutations linked to drug resistance were observed in 10 (3.2%) patients: 2 in the *rpoB* gene (1 His451Asp and 1 Ala411Val), and 8 in the *folP1* gene (2 Thr53Arg, 2 Thr53Ile, 3 Pro55Arg, and 1 Pro55Leu) (Tables 1 and 2). For the *gyrA* gene, 29 (9.2%) samples had mutations, but none were known AMR mutations. Fourteen (4.4%) samples presented the synonymous mutation Arg99Arg, related to subtype 3I (20). No multidrug resistance was observed. Nine of the mutations were found in relapse patients (*P* = 0,0014), and one mutation in the *folP1* gene was detected in a suspected treatment failure case (Table 1). Samples that failed to amplify by PCR or that yielded low-quality sequencing data, likely due to low bacteria DNA concentration, were labeled as “inconclusive” (Table 1).

**TABLE 2:** List of samples from leprosy cases with variants detected in genes investigated for AMR^*b*^

ID	Group	Clinical form	Regularity	Mutation (nucleotide change)							
				*rpoB*	*folP1*	*gyrA*	*gyrB*	*ctpC*	*ctpI*	*ribD*	*fadD9*
AM10	New case	LL	Yes	.	.	Arg99Arg	.	.	.	Thr144Ile	Ala162Ala
AM23	New case	LL	Yes	.	.	.	.	Gly667Arg	.	.	.
AM120	New case	LL	No	.	.	Arg99Arg	.	.	.	.	.
AM195	New case	LL	Yes	.	.	.	.	.			
AM207	New case	LL	Yes	.	.	Arg99Arg	.	.	.	.	Thr65Thr
AM223	New case	LL	Yes			.		.			.
AM251	New case	LL	Yes	.	.	.	.	Gly667Arg	.	.	.
AM261	New case	LL	No	.	.	Arg99Arg	.	.	.	.	.
AM316	New case	LL	Yes	.	.	.	.	Gly667Arg	.	.	.
AM337	New case	LL	Yes	.	.	.	.	.	.	.	Thr65Thr
AM358	New case	LL	No	.	.	Arg99Arg	.	.	.	.	.
AM411	New case	BL	No	.	.	Arg99Arg	.	.	.	.	.
AM422	New case	BL	No	.	.	Arg99Arg	.	.	.	.	.
AM12	Relapse	BL	Yes	.	.	Arg99Arg	.	.	.	.	Thr65Thr
AM20	Relapse	LL	Yes	.	.	.	.	Gly667Arg	.	.	.
AM27	Relapse	BL	No	.	**Thr53Arg** ^*a*^	.	.	.	.	Gly61Asp	.
AM30	Relapse	BL	Yes	.	.	.	.	.	.	.	Thr65Thr
AM33	Relapse	LL	No	.	.	.	.	.	.	.	.
AM62	Relapse	LL	No	.		.	.	.	.	.	.
AM73	Relapse	LL	No	.	.	.	Thr514Ala	.	.	.	.
AM91	Relapse	BL	No	.	.	.	.	.	.	.	Thr65Thr
AM97	Relapse	LL	No	.	.	.	.	.	.	.	.
AM98	Relapse	LL	No	**His451Asp** ^*a*^	.	.	.	.	.	.	.
AM105	Relapse	BL	Yes	.	.	.	.	.	.	.	.
AM169	Relapse	LL	Yes	.	.	.	.	.	.	.	.
AM181	Relapse	LL	Yes	.	.	.	.	Gly667Arg	.	.	.
AM232	Relapse	LL	Yes	.	.	.	.	.	.	Leu208Pro	.
AM254	Relapse	LL	No	.	.	.	.	Gly667Arg	.	.	.
AM260	Relapse	LL	No	.	.	Arg99Arg	.	.	.	.	.
AM267	Relapse	LL	Yes	.	.	.	.	.	.	.	Thr795Thr
AM268	Relapse	LL	Yes	.	.	.	.	.	.	.	.
AM292	Relapse	LL	No	**Ala411Val** ^*a*^	.	.	.	.	.	.	.
AM309	Relapse	LL	Yes	.	**Pro55Arg** ^*a*^	.	.	.	.	.	Asp520Asn
AM397	Relapse	LL	Yes	.	.	.	.	.	.	.	Ala162Ala Ile178Ile
AM434	Relapse	LL	No	.	**Pro55Leu** ^*a*^	.	.	.	.	Ser58Arg	Gly267Ser
AM581	Relapse	LL	Yes	.	**Thr53Ile** ^*a*^	.	.	.	.	.	.
AM959	Relapse	ND	Yes	.	**Pro55Arg** ^*a*^	.	.	.	.	.	.
AM1021	Relapse	ND	Yes	.	**Thr53Ile** ^*a*^	.	.	.	.	.	.
AM1148	Relapse	ND	No	.	**Thr53Arg** ^*a*^	.	.	.	.	.	.
AM147	Susp. of failure	LL	No	.	.	.	.	.	.	.	Thr65Thr
AM220	Susp. of failure	LL	Yes	.	.	.	.	.	.	.	Thr65Thr
AM423	Susp. of failure	ND	Yes	.	.	.	.	.	.	.	Thr65Thr
AM965	Susp. of failure	LL	Yes	.	**Pro55Arg** ^*a*^	.	.	.	.	.	.

^*a*^ Mutation previously related to AMR.

^*b*^ BL, borderline lepromatous; ID, sample name; LL, lepromatous lepromatous; ND, not determined; Susp. of failure, suspect of failure; ".", No mutation detected.

Mutation screening of the *gyrB*, *ctpC, ctpI, ribD*, and *fadD9* genes was performed on 163 (50.7%) of the samples. Of these, 25 (15.3%) showed mutations, with *fadD9* exhibiting the most variability (6 mutations in 15 [9.1%] strains). The *ctpC* gene had the variant Gly667Arg in six (3.6%) samples, mostly from relapse patients (Table 2). The *fadD9* gene included two nonsynonymous mutations (Gly267Ser and Asp520Asn), both in dapsone-resistant strains. In screening for the *fadD9* gene, partial amplification occurred in 60 (36.8%) samples, where not all 3 PCR products were amplified. The *ribD* gene had four nonsynonymous mutations, two of which were novel (Thr144Ile and Phe208Pro), and two previously reported (Gly61Asp and Ser58Arg) (15), found in two relapse samples with dapsone resistance. No mutations were identified in the *ctpI* gene across the analyzed samples. No mutations were identified in the *ctpl* gene. Eight samples had mutations in more than one gene, with six from the relapse group (Table 2). In total, 43 out of 315 (13.6%) samples exhibited mutations in the evaluated genes, indicating significant genetic diversity and potential AMR implications for *M. leprae* strains in Amazonas.

The genotype of *M. leprae* was determined in 147 (90.2%) of the 163 samples. Genotype 4 was the most common (117 samples, 79.6%), followed by genotype 3 (14 samples, 8.6%). Two samples exhibited a mutation at position 2935685 related to TN strain (AL450380.1), placing them between genotypes 1 and 2.

## DISCUSSION

Historically, Amazonas was one of the first Brazilian states to implement MDT as a standard treatment, following the identification of sulfone resistance in patients at Colônia Antônio Aleixo (21). Subsequent studies revealed resistance to rifampicin, ofloxacin, and dapsone, including cases of multidrug resistance and primary resistance (6, 7). Systematic monitoring of relapse cases began in 2012 with the establishment of a molecular biology lab at AMF, later expanding to include suspected therapeutic failures, uncontrolled leprosy reactions, and new multibacillary cases. Results have been reported to the Ministry of Health and WHO.

The first WHO surveillance report (2009–2015) revealed concerning resistance rates in Brazil: rifampicin (9.1%), dapsone (12.1%), and ofloxacin (3%) (22). Brazil’s *M. leprae* Resistance Surveillance Plan, initiated in October 2018, aimed to expand national sampling. From 2018 to 2022, the overall prevalence was 2.5% (61/2,463), with coverage at 0.9% for new MB cases and 12.3% for relapse cases, highlighting the necessity for broader testing (9).

Our study detected a 3.2% (10/315) prevalence of MDT drug resistance in Amazonas, slightly above the national average but lower than in some other regions, including a hyperendemic area in Pará (23). Most resistant strains were from relapse patients, clinically classified as borderline-lepromatous or lepromatous, consistent with other studies (22–24). High bacillary load in these patients increases the risk of selecting resistant strains (25). Half of the resistant samples had a history of irregular treatment. No primary resistance was detected in our sample, though it has been previously reported (7).

Of the two variants detected in the *rpoB* gene, His451Asp is known to determine rifampicin AMR (26). The second variant, Ala411Val, was not previously reported and is yet to be demonstrated as causal of AMR *in vivo*. The functional impact of this mutation on the protein was assessed using the HARP platform (Hansen’s Disease Antimicrobial Resistance Profiles) (27), a database of structural impacts of systematic missense mutations in drug targets of *M. leprae,* which classified its impact as low. The low frequency of rifampicin-resistant samples observed in our study contrasts with findings from other countries such as China (28) and India (29).

AMR-related mutations in the *gyrA* gene were absent, diverging from national data showing increased ofloxacin resistance post-COVID-19. The highest frequency of AMR mutations was observed in the *folP1* gene (2.5%), consistent with findings from countries such as Malaysia (30) and the Ivory Coast (31). Mutations were detected at codon 53 (Thr53Arg and Thr53Ile) and 55 (Pro55Arg and Pro55Leu), confirming the presence of diverse resistant *M. leprae* strains in Amazonas. Notably, dapsone has been used for over 80 years in both monotherapy and as part of MDT for PB and MB cases in the region (21). Seven resistant samples were from relapse cases, and one was suspected of treatment failure. The circulation of dapsone-resistant strains may increase pressure on rifampicin and clofazimine, raising the risk of further resistance. Geographically, mutations were primarily found in Manaus, although cases were also detected in other municipalities across Amazonas.

Clinical treatment failures are often associated with intermediate or high resistance levels, as determined by Mouse Footpad (MFP) assays, although mutations may or may not be present in cases of intermediate resistance (30). This suggests that these isolates may harbor mutations in other genes (10). To investigate potential AMR-related mutations, we analyzed 5 additional genes in 163 patients from the AMF AMR surveillance (2012 and 2018): *ctpC* (3.6%), *ctpI* (0%), *fadD9* (9.1%), *ribD* (2.4%), and *gyrB* (0.6%). Notably, mutations in *ctpC* and *ctpI* have been linked to intermediate rifampicin resistance (10), while *gyrB* mutations have been previously identified in dapsone-resistant *M. leprae* (15, 32, 33). The Thr514Ala mutation in the *gyrB* gene has not been previously described or experimentally assessed; however, analysis via the HARP platform suggests it moderately affects the gene function. This study highlights the genetic diversity of *M. leprae* strains in Amazonas. While mutations in these genes do not confirm AMR and may represent natural genotypic variations rather than being directly associated with antimicrobial resistance, they indicate strong selective pressure and highlight the need for further investigation of their role in therapeutic response (15). These genes have not been previously analyzed in Amazonas, and few studies have explored them in diverse global populations (15, 34, 35). The use of next-generation sequencing has advanced AMR monitoring, as demonstrated in studies conducted in China, Comoros, and Brazil (34–36), enhancing the identification of resistant subpopulations and improving data accuracy.

For the first time in this sample collection, we identified the predominant SNP type 4, consistent with previous findings in Brazilian samples (15). Two samples showed mutations associated with SNP type 1 or 2, which requires further confirmation. Although 315 biopsy samples were included, the sample may not fully represent the diversity of *M. leprae* strains across all regions of Amazonas or other endemic areas outside the state. The predominance of cases (92.6%) from Manaus suggests an urban bias. Additionally, the clinical significance of some observed mutations remains unclear, and further research, including functional assays or animal models, is needed to assess their impact on drug resistance and treatment outcomes.

In conclusion, the mutations identified in this study highlight the importance of expanding AMR monitoring in *M. leprae.* This will provide more comprehensive data on mutations in alternative genes and emphasize the need for further investigations into new AMR mechanisms in leprosy. Our findings offer insights that could inform treatment and control strategies in Amazonas and similar endemic regions worldwide. Understanding local resistance patterns and genetic diversity is essential for optimizing treatment regimens and improving surveillance to address emerging drug resistance.

## SUPPLEMENTAL MATERIAL

Supplemental material aac.01550-24-s0001.xlsx Tables S1 to S3.

ASM does not own the copyrights to Supplemental Material that may be linked to, or accessed through, an article. The authors have granted ASM a non-exclusive, world-wide license to publish the Supplemental Material files. Please contact the corresponding author directly for reuse.
